# Central-line–associated bloodstream infections and central-line–associated non-CLABSI complications among pediatric oncology patients

**DOI:** 10.1017/ice.2022.91

**Published:** 2023-03

**Authors:** Aml S. Kelada, Timothy B. Foster, Gregory C. Gagliano, Sarah Worley, Anne Tang, Venkatraman A. Arakoni, Charles B. Foster

**Affiliations:** 1 Department of Pediatric Hematology Oncology and Blood and Marrow Transplantation, Cleveland Clinic, Cleveland, Ohio; 2 Center for Pediatric Infectious Diseases, Cleveland Clinic, Cleveland, Ohio; 3 Quality and Patient Safety Institute, Cleveland Clinic, Cleveland, Ohio; 4 Department of Quantitative Health Science, Cleveland Clinic, Cleveland, Ohio

## Abstract

**Objective:**

To assess central venous catheter (CVC) harm in pediatric oncology patients, we explored risks for central-line–associated bloodstream infections (CLABSIs) and central-line–associated non-CLABSI complications (CLANCs).

**Design:**

Retrospective cohort study.

**Setting:**

Midwestern US pediatric oncology program.

**Patients:**

The study cohort comprised 592 pediatric oncology patients seen between 2006 and 2016.

**Methods:**

CLABSIs were defined according to Centers for Disease Control and Prevention (CDC)/National Health Safety Network (NHSN) definitions. CLANCs were classified using a novel definition requiring CVC removal. Patient-level and central-line–level risks were calculated using a negative binomial model to adjust for correlations between total events and line numbers.

**Results:**

CVCs were inserted in 62% of patients, with 175,937 total catheter days. The inpatient CLABSI and CLANC rates were 5.8 and 8.5 times higher than outpatient rates. At the patient level, shared risks included acute myeloid leukemia (AML) and age <1 year at diagnosis. At the line level, shared risks included age <1 year at diagnosis, non-mediports, and >1 lumen. AML was a CLABSI-specific risk. CLANC-specific risks included non–brain-tumor diagnosis, younger age at diagnosis or central-line placement, and age <1 year at diagnosis or line placement. Multivariable risks were for CLABSI >1 lumen and for CLANC age <1 year at placement.

**Conclusions:**

Among patients with CVCs, CLABSI and CLANC rates were similar, higher among inpatients than outpatients. For both CLABSIs and CLANCs, infants and patients with AML were at higher risk. In both univariate and multivariate models, lines with >1 lumen were associated with CLABSIs and placement during infancy with CLANCs.

Among pediatric oncology patients, central-line–associated bloodstream infections (CLABSIs) are an important healthcare-associated complication, responsible for significant cost, morbidity, and mortality.^1,2^ In contrast to inpatient CLABSIs, relatively little effort has been devoted to preventing outpatient CLABSIs or central-line–associated non-CLABSI complications (CLANCs).^3–16^ A comprehensive analysis of harm associated with CVC use might define risk factors, inform CLABSI and CLANC prevention initiatives, and guide CVC selection.

To define risk factors and rates of CVC-associated harm that occurred over a 10-year period in children and young adults undergoing cancer treatment, we characterized CVC use and identified all CLABSIs and CLANCs, stratifying events by whether they occurred in the inpatient or outpatient setting. A novel aspect of this study was the development of a measure analogous to CLABSI for quantifying central-line–associated non-CLABSI complications.

## Methods

### Patient population and study design

We retrospectively reviewed CVC use in 592 consecutive children and young adults (aged ≤24 years) with cancer who initiated care at Cleveland Clinic Children’s Department of Pediatric Hematology Oncology and Blood and Marrow Transplantation between January 2006 and March 2016. In this regard, the population reflects the distribution of patients cared for in a general pediatric oncology program. Demographic and clinical outcomes data were collected on patients having an oncologic diagnosis and placement of a CVC. The primary *International Classification of Disease for Oncology* (ICD-O) diagnostic code was used to assign diagnostic categories: leukemia or lymphoma, soft-tissue tumor and sarcoma, brain tumor, or other malignancy.^17^ Patients with leukemia or lymphoma were stratified according to whether they had acute myeloid leukemia (AML) or non-AML leukemia or lymphoma. Data were collected through December 31, 2016.

### Central-line data

A current procedural terminology (CPT) code–based algorithm, supplemented by manual chart review, identified CVC insertion and removal procedures. We documented all CVCs, along with catheter type, number of lumens, insertion, and removal (or last contact) date, and removal indication. CVCs in place for <1 day were excluded. CVCs were categorized as tunneled (eg, Broviac or Hickman), apheresis (large bore typically multilumen tunneled or not tunneled), nontunneled (eg, peripherally inserted central catheters or internal jugular), or mediport (ie, implanted subcutaneous).

### Central-line–associated bloodstream infection (CLABSI) data

CLABSIs were defined according to Centers for Disease Control and Prevention/National Healthcare Safety Network (CDC/NHSN) definitions and were further subclassified if mucosal barrier injury (MBI) was associated. CLABSIs were considered to have occurred in the outpatient setting if the first positive blood culture occurred >48 hours after hospital discharge or <48 hours after admission. CLABSIs were considered to have occurred in the inpatient setting if the first positive blood culture occurred >48 hours after admission or <48 hours after hospital discharge. To identify CLABSIs, we reviewed complete blood-culture data.

### Central-line–associated non-CLABSI complication (CLANC) data

CLANCs were defined as non-CLABSI complications that resulted in CVC removal due to malfunction, malposition, dislodgement, breakage, thrombosis, exit site problem, contamination or need for relocation (Table 1). Lines removed at the time of a CLABSI were excluded from the CLANC definition. CLANCs were considered to have occurred in the outpatient setting if the patient was not hospitalized in the calendar day preceding the event. CLANCs were considered to have occurred in the inpatient setting if the event occurred while the patient was hospitalized, or if the patient was admitted the calendar day preceding the event. To identify CLANCs, we reviewed charts to determine indications for line removal.

**Table 1.** Indication for Central Line Removal and Metric Description for Central-Line–Associated Non-CLABSI Complications (CLANCs) Occurring in Pediatric and Young Adult Oncology Patients

Reason for Removal	Metric Description	No. of Lines (n=82), No. (%)
Breakage-CLANC	Catheter removed due to broken, fractured, or cracked tubing or access port, or leakage. Does not include successfully repaired catheters.	16 (20)
Contamination-CLANC	Catheter removed due to concern for loss of sterility, such as from fecal soilage, injection of foreign material, or failure to use a protective cap or needless access device.	2 (2.4)
Dislodgement-CLANC	Catheter accidentally or intentionally pulled out either fully or partially by the patient or caregiver, accidental catheter removal by staff, or failure of catheter securement resulting in catheter dislodgement.	14 (17)
Exit Site Problem-CLANC	Catheter removed due to irritation, breakdown, inflammation or infection at the exit site.	8 (9.8)
Malfunction-CLANC	Catheter removed due to occlusion, inability to flush, sluggish or no blood return. Does not include catheters successfully treated with medications to clear an occlusion.	22 (27)
Malposition-CLANC	Catheter removed due to migration, coiling or kinking of the proximal tip. Does not include catheters where the tip is in the wrong location due to dislodgement.	16 (20)
Relocation-CLANC	Catheter removed and relocated to a new site due to inappropriate placement location or patient discomfort.	3 (3.6)
Thrombosis-CLANC	Catheter removed due to thrombosis or clot. Does not include late vascular occlusion events recognized after catheter removal.	1 (1.2)

Note. CLABSI, central-line–associated bloodstream infection; CLANC, central-line–associated non-CLABSI complication.

### Inpatient and outpatient event and rate data

A computer program in Python was written to input complete inpatient admission and discharge data, CLABSI data and central-line–specific CLANC data. Analyses were performed at the patient level and the line level. For patient-level analyses, we calculated the total number of lines, catheter days (days when at least 1 line was in place), line days (sum of all lines in place on each catheter day), line density (line days/catheter days), lumen days (similar to line days but counting every lumen), lumen density (lumen days/catheter days), number of CLABSIs, and number of CLANCs. For all catheter types, we used the insertion and removal (or last contact) date to calculate catheter and line days. Events were classified as inpatient or outpatient, and rates were calculated for each setting. For line-level analyses, we calculated the number of line days, lumen days, CLABSIs and CLANCs for each line. Inpatient and outpatient overall event rates were calculated summing the CLABSI and CLANC events. For line-level analyses, if >1 line was present at the time of a CLABSI, the event was randomly assigned to one of the lines.

### Statistical analysis

Data were described using medians and quartiles for continuous variables and counts and percentages for categorical variables. Sample sizes for individual variables reflect missing data. To estimate patient-level and central-line–level event rates with 95% confidence intervals and to assess their associations with demographic and clinical risk factors, separate negative binomial regression models were used for each risk factor and event type, using the event count as the outcome and the log of the catheter or line days for the offset of the patient-level and central-line–level analyses, respectively. For multivariate analysis, a logistic regression model was used where all lines were treated independently. For the central-line–level analysis, the primary analysis treated each line independently with a sensitivity analysis conducted on only the first lines by date of placement. All tests were 2-tailed and were performed at a significance level of 0.05. SAS version 9.4 software (SAS Institute, Cary, NC) was used for all analyses.

## Results

### Patient population

Of the 592 pediatric oncology patients, 366 (62%) had at least 1 CVC. Among these patients (Table 2), the initial oncology visit occurred at a median age of 7 years (range, 0–24). The most common primary oncology diagnoses were leukemia or lymphoma (n = 147, 40%), soft-tissue tumors and sarcomas (n = 123, 34%) and brain tumors (n = 80, 22%), and AML patients accounted for 18 cases (4.9%). The total number of central lines per patient ranged from 1 to 13, with 222 (61%) having 1 central line, 78 (21%) having 2 central lines, and 66 (18%) having 3 or more central lines.

**Table 2.** Demographic Characteristics of Pediatric and Young Adult Oncology Patients with Central Lines

Characteristic	No. of Patients (n=366)
Age at diagnosis, median y (min, max)	7 (0, 24)
Age at diagnosis, median y (IQR)	7 (3–13)
**Age at diagnosis, no. (%)**	
<1 y	26 (7.1)
1–2 y	60 (16)
3–11 y	146 (40)
12–17 y	115 (31)
18–24 y	19 (5.2)
**Sex, no. (%)^a^**	
Female	161 (44)
Male	204 (56)
**Diagnosis, no. (%)**	
Leukemia/Lymphoma	147 (40)
Soft-tissue tumors	123 (34)
Brain tumors	80 (22)
Others	16 (4.4)
**AML, no. (%)**	
No	348 (95)
Yes	18 (4.9)
**Total CVC, no. (%)**	
1	222 (61)
2	78 (21)
3 or more	66 (18)
Total CVC, median (min, max)	1 (1, 13)

Note. AML, acute myeloid leukemia; CVC, central venous catheter.

a Data not available for 1 study participant.

### Central-line details

Demographic and clinical characteristics of the 650 CVCs are described in Supplementary Table 1 (online). The median age at placement was 7 years. The most common CVCs were mediports (n = 375, 58%) and tunneled lines (n = 168, 26%), with apheresis and nontunneled CVCs accounting for 54 CVCs (8.3%) and 53 CVCs (8.2%), respectively. Bone-marrow transplant patients accounted for 42 lines (6.5%). The median duration of line placement was 152 days: 17 days for inpatients and 122 days for outpatients.

### Central-line–related events and removal indications

We identified 192 CVC-related harm events: 110 CLABSIs and 82 CLANCs. Among CVCs with CLABSIs, 90 (91%) had 1 CVC-related harm event, 7 (7%) had 2 harm events, and 2 (2%) had 3 harm events. CLANCs occurred in 82 CVCs (13%) (Table 1). The following indications for removal were observed: 22 (27%) due to malfunction, 16 (20%) due to breakage, 16 (20%) malposition, 14 (17%) due to dislodgement, 8 (9.8%) due to an exit-site complication, 3 (3.7%) due to the need for relocation, 2 (2.4%) due to contamination, and 1 (1.2%) due to thrombosis. Overall, 146 CVCs (22%) were removed due to a line-related adverse event, 57 (39%) were removed due to a CLABSI, 82 (56%) were removed due to a CLANC, and 7 (4.8%) were removed due to culture-negative sepsis.

### Catheter days, central-line days, and unadjusted event rates

CVC use and harm events for inpatients and outpatients are described in Table 3. There were 175,937 catheter days and 177,448 line days. Most patients had a single CVC (line density, 1.01). Multilumen catheters were more common among inpatients (lumen density, 1.57) than outpatients (lumen density, 1.11). The unadjusted CLABSI rates per 1,000 catheter days were 0.63 total: 2.48 in the inpatient setting and 0.43 in the outpatient setting. The unadjusted CLANC rates per 1,000 catheter days were 0.47 total: 2.30 in the inpatient setting and 0.27 in the outpatient setting.

**Table 3.** Comparison of Outpatient and Inpatient Catheter Days, Line Densities and Unadjusted Harm Rates for Pediatric and Young Adult Oncology Patients with Central Lines

Measure	Outpatient	Inpatient	Total
Line days^a^	160,023	17,425	177,448
Catheter days^b^	158,992	16,945	175,937
Line density^c^	1.01	1.03	1.01
Lumen density^d^	1.11	1.57	1.15
CLABSIs	68	42	110
MBI-CLABSIs	16	20	36
CLANCs	43	39	82
CLABSI rate^e^	0.43	2.48	0.63
MBI-CLABSI rate^e^	0.10	1.18	0.20
CLANC rate^e^	0.27	2.30	0.47
All event rate^e^	0.70	4.78	1.09

Note. CLABSI, central–line–associated bloodstream infection; CLANC, central-line–associated non-CLABSI complication. MBI, mucosal barrier injury.

a Line days are the sum of all central venous catheters in place on each catheter day.

b Catheter days are calendar days when at least 1 central venous catheter was in place.

c Line density is the number of line days/catheter days.

d Lumen density is the sum of the lumens for all central venous catheters in place on each catheter day/catheter days.

e Incidence rates are per 1,000 catheter days.

### Patient-level adjusted event rates

A negative binomial model was used to estimate the mean event rates at the patient level and the line level. At the patient level (Table 4), the adjusted incidence rates per 1,000 patient catheter days were as follows: all events, 1.5 (95% confidence interval [CI], 1.1–1.9); CLABSIs, 0.79 (95% CI, 0.59–1.1); and CLANCs, 0.59 (95% CI, 0.42–0.84). The incidence rates for each event type were higher among inpatients than outpatients.

**Table 4.** Patient-Level Adjusted Incidence Rates for All Events, CLABSIs and CLANCs among All Patients, Inpatients and Outpatients

Event Type	Incidence Rate per 1,000 Catheter Days^a^ (95% CI)		
	All patients (n=366)	Inpatients (n=334)	Outpatients (n=354)
All events	1.5 (1.1–1.9)	3.9 (2.9–5.3)	0.93 (0.68–1.3)
CLABSIs	0.79 (0.59–1.1)	2.2 (1.5–3.2)	0.53 (0.37–0.75)
CLANCs	0.59 (0.42–0.84)	1.9 (1.3–2.9)	0.29 (0.20–0.42)

Note. CLABSI, central-line–associated bloodstream infection; CLANC, central-line–associated non-CLABSI complication.

a Catheter days, calendar days when at least 1 central venous catheter was in place;

### Patient-level event rates and harm risk factors

Patient-level incidence rate ratios and 95% CIs of each potential risk factor for CLABSIs, CLANCs, and both are shown in Supplementary Table 2 (online). For all events, an increased incidence rate was associated with a higher number of central lines per patient, female sex, AML, a non–brain-tumor diagnosis, younger age at diagnosis (per 1 year), and age at diagnosis <1 year. An increased risk for CLABSI was associated with a higher number of lines per patient, AML, and age at diagnosis <1 year. An increased risk for CLANC was associated with higher number of lines per patient, AML, a non–brain-tumor diagnosis, younger age at diagnosis, and age at diagnosis <1 year.

### Line-level adjusted event rates

Examining each line independently (Table 5), the adjusted incidence rates per 1,000 line days were 3.8 for all events (95% CI, 2.8–5.2), 1.6 for CLABSIs (95% CI, 1.1–2.3), and 2.3 for CLANCs (95% CI, 1.4–3.7). The incidence rates for CLABSIs were greater in the inpatient setting than the outpatient setting. Incidence rates for CLANCs were also higher in the inpatient setting than the outpatient setting, although the confidence intervals overlapped.

**Table 5.** Central Line-Level Adjusted Incidence Rates for All Events, CLABSIs, and CLANCs among All Patients, Inpatients, and Outpatients for all Central Lines, Treated Independently (n = 650)

Event Type	Incidence Rate per 1,000 Central-Line Days (95% CI)		
	All Central Lines (n = 650)	Inpatient Central Lines (n = 582)	Outpatient Central Lines (n = 553)
All events	3.8 (2.8–5.2)	4.8 (3.8–6.1)	2.2 (1.5–3.4)
CLABSIs	1.6 (1.1–2.3)	2.4 (1.8–3.3)	1.1 (0.67–1.8)
CLANCs	2.3 (1.4–3.7)	2.2 (1.6–3.1)	1.0 (0.51–2.0)

Note. CLABSI, central-line–associated bloodstream infection; CLANC, central-line–associated non-CLABSI complication.

### Central-line–level event rates and harm risk factors

Among all lines (Table 6), the overall adverse event risk increased with higher line rank order, AML, a non–brain-tumor diagnosis, younger age at diagnosis, age at diagnosis <1 year, younger age at placement (per 1 year), age at placement <1 year, not tunneled status, non-mediport, and >1 lumen. The CLABSI risk was increased among patients with AML, non-brain tumor diagnosis, age at diagnosis <1 year, not tunneled status, non-mediport, and with >1 lumen. The CLANC risk increased with younger age at diagnosis, age at diagnosis <1 year, younger age at placement, age at placement <1 year, nontunneled status, nonmediport, and >1 lumen.

**Table 6.** Comparison of Incidence Rate Ratios and Risk Factors for CLABSIs, CLANCs and All Events for all Central Lines Treated Independently (n=650)

Risk Factor	Comparison	CLABSI Incidence Rate Ratio (95% CI)	*P* Value	CLANC Incidence Rate Ratio (95% CI)	*P* Value	All Events Incidence Rate Ratio (95% CI)	*P* Value
Age at diagnosis	Per 1 year older	0.95 (0.91–1.00)	.061	0.83 (0.78–0.89)	<.001	0.90 (0.87–0.94)	<.001
	< 1 year vs older	4.8 (2.1–11.1)	<.001	29.7 (13.6–65.1)	<.001	11.6 (6.1–22.0)	<.001
Age at placement	Per 1 year older	0.96 (0.92–1.01)	.13	0.83 (0.78–0.89)	<.001	0.91 (0.87–0.95)	<.001
	< 1 year vs older	2.8 (0.96–8.4)	.059	31.0 (10.3–93.0)	<.001	10.1 (4.3–23.8)	<.001
Diagnosis	AML vs all others	4.0 (1.5–10.5)	.005	2.7 (0.66–10.6)	.17	3.2 (1.4–7.5)	.008
	AML vs Non-AML leukemia/lymphoma	3.7 (1.3–10.6)	.017	2.5 (0.63–9.6)	.20	2.7 (1.1–6.7)	.028
	AML vs brain tumors	7.4 (2.2–24.5)	.001	8.2 (1.6–41.0)	.01	7.0 (2.5–19.8)	<.001
	All others vs brain tumors	1.6 (0.32–7.9)	.58	18.1 (3.4–96.9)	<.001	6.7 (2.1–21.7)	.002
Sex	Female vs male	1.2 (0.62–2.3)	.60	2.1 (0.89–5.1)	.089	1.5 (0.86–2.6)	.16
Central-line insertion rank order	Per 1 additional central line	1.2 (0.97–1.5)	.09	1.3 (0.99–1.7)	.06	1.2 (1.04–1.5)	.015
CVC type	>1 lumen vs 1 lumen	11.0 (6.8–17.6)	<.001	10.8 (6.0–19.4)	<.001	10.8 (7.2–16.0)	<.001
	Not tunneled vs tunneled	3.3 (1.2–9.2)	.022	5.2 (1.5–17.6)	.009	4.0 (1.8–9.3)	.001
	Non-mediport vs mediport	12.3 (7.7–19.5)	<.001	19.8 (10.9–35.9)	<.001	15.1 (10.3–22.0)	<.001
	Non-tunneled vs mediport	12.3 (4.5–33.2)	<.001	22.3 (7.0–71.2)	<.001	15.7 (7.2–34.5)	<.001
	Tunneled vs mediport	12.3 (7.5–20.1)	<.001	20.7 (10.9–39.3)	<.001	15.4 (10.3–23.2)	<.001
	Apheresis catheter vs mediport	12.2 (4.8–30.8)	<.001	22.9 (7.5–69.6)	<.001	15.8 (7.5–33.3)	<.001

Note. AML, acute myeloid leukemia; CLABSI, central-line–associated bloodstream infection; CLANC, central-line–associated non-CLABSI complication; CVC, central venous catheter.

To characterize CLANC risk by patient age, we examined line usage and reason for removal for infants (Supplementary Table 3 online) and older patients (Supplementary Table 4 online). Tunneled CVCs were used more frequently in infants than in older patients: 33 (69%) of 48 infants versus 135 (22%) of 602 older patients (*P* < .001). For tunneled CVCs, dislodgement was the removal indication for 6 (18%) of 33 infants versus 6 (4.4%) of 135 older patients (*P* = .014). The next most common indication for removal in infants was malposition, which occurred in 5 (15%) of 33 infants versus 3 (2.2%) of 135 older patients (*P* = .008). Among the 11 infants with mediports, 2 CLANCs (18%) were due to malfunction and 1 each was due to malposition and exit-site problems, whereas in 364 older patients, malfunction was most common reason for removal (n = 8, 2.2%; *P* = .031).

### First central-line event rates and harm risk factors

Because harm events were less frequent among first central lines, we examined risk factors for CVC-associated harm in these central lines (Supplementary Table 5 online). Factors associated with first central-line CLABSI included AML, age at diagnosis <1 year, age at placement <1 year, nontunneled status, non-mediport and >1 lumen. Factors associated with a first central-line CLANC included younger age at diagnosis, age at diagnosis <1 year, younger age at placement, age at placement <1, nontunneled status, and non-mediport.

### Mucosal barrier injury–associated CLABSI rates and risk factors

MBI-CLABSIs were analyzed separately because they may have distinct risk factors.^18–20^ Of 110 total CLABSIs, 36 (33%) were MBI-CLABSIs and 20 (56%) of these occurred among inpatients and 16 (44%) occurred among outpatients (Table 3). The MBI-CLABSI incidence rate per 1,000 catheter days was 0.29 overall (95% CI, 0.18–0.48): 1.1 (95% CI, 0.66–1.8) in the inpatient setting and 0.15 (95% CI, 0.070–1.31) in the outpatient setting. Patient-level MBI-CLABSI risk factors included a higher lifetime number of CVCs, female sex, AML, and age at diagnosis <1 year (Supplementary Table 6 only). Increased line-level MBI-CLABSI risk was associated with AML, age at diagnosis <1 year, non-mediport, and >1 lumen (Supplementary Table 7 online).

### Multivariable model

In the multivariable model, using the independent variables of AML, age at placement <1 year, mediport, >1 lumen, and line rank order, CLABSI risk was associated with >1 lumen (OR, 2.7; 95% CI, 1.4–5.5; *P* = .005), and CLANC risk was associated with age <1 year at placement (OR, 7.5; 95% CI, 3.8–14.8; *P* < .001).

## Discussion

We characterized CLABSIs and non-CLABSI CVC harm events in 366 children and young adults receiving cancer treatment; inpatient CLABSIs accounted for 22% of the events. Also, 38% of CLABSIs occurred among inpatients, even though patients spent 90% of their time with a CVC in the outpatient setting. The inpatient CLABSI rate was 5.8 times higher than the outpatient CLABSI rate. Similarly, the inpatient CLANC rate was 8.5 times higher than the outpatient rate, with event totals being similar in the 2 settings. The relative frequency of CLABSIs and CLANCs were similar. The inpatient unadjusted CLABSI and CLANC rates were 2.48 and 2.30 per 1,000 catheter days, respectively. The outpatient CLABSI and CLANC rates were 0.43 and 0.27 per 1,000 catheter days, respectively.

A novel aspect of this study was the development of a measure analogous to CLABSI for quantifying central-line–associated non-CLABSI complications resulting in line removal, referred to here as CLANCs. Other investigators have explored nonelective CVC removal; however, the incidence and risk factors have received little attention.^7,8,21–25^ In this study, the most common CLANC types were malfunction, malposition, breakage, and dislodgement. Understanding the types of events and populations where catheter loss occurs may inform CVC selection and quality improvement initiatives. An intriguing finding from this study is that overall catheter harm rates were lowest in first lines and increased with each subsequent line.

Because catheter choice is a modifiable risk factor, it is important to recognize that CLABSI and CLANC risks may be associated with specific CVC types. Compared with tunneled catheters, implanted mediports had lower risk for both CLABSI and CLANCs. Similarly, CLABSI and CLANC risk was increased for CVC with >1 lumen. In the multivariate analysis, there was a strong association between lines with >1 lumen and CLABSIs. The literature provides confirmatory evidence that catheter choice is an important risk determinant for CVC-associated harm.^5,23,25–31^

Infants were at high risk for both CLABSIs and CLANCs. Compared to older children, infants were less likely to have mediports and were more likely to have tunneled CVCs. Infants were prone to specific CLANCs, especially line dislodgement. CLANCs, especially malfunction, occurred at increased frequency in infants with mediports. Because our sample size was small, our data did not provide preferential support for the use of mediports in this age group. Nonetheless, specific efforts need to be taken in infants and toddlers to mitigate the risk of CLABSIs and CLANCs. These might include directing the catheter tubing away from the diaper to avoid fecal contamination and implementation of measures to prevent tugging, chewing or sucking on the tubing, and other factors that may contribute to dislodgement, breakage, or infection.

AML patients were at high risk for developing both CLABSIs and CLANCs. Perhaps reflecting the need for dose-intensive chemotherapy and the anticipated need for prolonged inpatient care, 89% of lines in our AML patients were double-lumen and tunneled CVCs, the catheter types associated in the overall population with increased CLABSI and CLANC risk. The small number of AML patients in our study limited our ability to draw conclusions about whether line choice specifically contributed to CLABSIs in this subpopulation. Because AML patients are at increased risk for bacteremia following dose-intensive induction chemotherapy, especially with oral mucosal bacteria (eg, *viridans* group streptococci), it is possible that episodes of bacteremia in this population are related to mucosal barrier injury.^19,32,33^ In this regard, in the full population, MBI-CLABSI–specific risk factors at the patient and central-line levels included AML and aged <1 year at diagnosis. In addition, at the central-line–level, MBI-CLABSI risk was higher for patients with non-mediports and those with lines with >1 lumen.

Although we comprehensively examined risk factors for both CLABSIs and CLANCs, our study had several limitations. We did not examine the impact of specific line-care practices believed to impact event rates, such as insertion and maintenance bundle care, line entry frequency, or use of parenteral nutrition.^16,34–36^ Increased adherence to best-practice guidelines, as has been achieved by many hospitals in recent years, may narrow risk differences between catheter types.^37,38^ Similarly, this study was completed during a period when our hospital was not routinely using antibacterial prophylaxis for patients with AML.^39^

Extrapolating conclusions from this small single-center cohort study to nongeneral pediatric oncology populations should be done with care. Only a minority of lines in this study were placed in bone marrow transplant patients, and line requirements differ for this population. Finally, we used a pragmatic definition to identify CLANCs and included only events identified as having directly contributed to line removal. In this regard, the CLANC definition did not include temporary harm events corrected by line repair or manipulation, catheters successfully treated with medications to clear an occlusion, or events recognized at a delayed period following catheter removal, such as late vascular occlusion.^40^

The high frequency of CVC harm events in pediatric oncology patients underscores the need for additional quality-improvement initiatives. Several factors, however, hinder the ability of hospitals to expand CVC harm surveillance beyond inpatient CLABSIs. These factors include accessing data on outpatient catheter days, lack of standard definitions for noninfectious CVC complications, lack of surveillance resources, and the lack of a standardized hospital or national reporting infrastructure.

In conclusion, among pediatric oncology patients with CVCs, inpatient CLABSIs accounted for only 22% of harm events. Half of all CLABSIs occurred among outpatients, and central-line–associated non-CLABSI complications occurred at rates similar to CLABSIs. Infants and patients with AML were at especially high risk for developing both CLABSIs and CLANCs. In multivariable analysis, lines with >1 lumen were associated with increased CLABSI risk and lines placed during infancy were associated with increased CLANC risk.

## Supplementary material

For supplementary material accompanying this paper visit https://doi.org/10.1017/ice.2022.91. click here to view supplementary material
