# Supplementary material for: Central-line–associated bloodstream infections and central-line–associated non-CLABSI complications among pediatric oncology patients
Source: Infect Control Hosp Epidemiol. 2022 Apr 27;44(3):377–83. doi: 10.1017/ice.2022.91 (PMC10015264; doi:10.1017/ice.2022.91)
Supplement: Supplementary file 1 [file S0899823X22000915sup001.zip › S0899823X22000915supp001.docx]

| Supplemental Table 4. Indication for Central Line Removal for Pediatric Oncology and Young Adult Patients > 1 year at Age of Placement Stratified by Line Type | | | | |
| --- | --- | --- | --- | --- |
| Factor | Tunneled (n=135) | Mediport (n=364) | Apheresis (n=52) | Non-tunneled (n=51) |
| Reason for line removal |  |  |  |  |
| CLABSI | 26 (19) | 20 (5.5) | 3 (5.8) | 4 (7.8) |
| Breakage-CLANC | 8 (5.9) | 4 (1.1) | 1 (1.9) | 0 (0) |
| Contamination-CLANC | 1 (0.74) | 0 (0) | 0 (0) | 0 (0) |
| Dislodgement-CLANC | 6 (4.4) | 0 (0) | 1 (1.9) | 1 (2.0) |
| Exit Site-CLANC | 3 (2.2) | 4 (1.1) | 0 (0) | 0 (0) |
| Malfunction-CLANC | 5 (3.7) | 8 (2.2) | 3 (5.8) | 2 (3.9) |
| Malposition-CLANC | 3 (2.2) | 5 (1.4) | 1 (1.9) | 0 (0) |
| Relocation-CLANC | 2 (1.5) | 0 (0) | 0 (0) | 0 (0) |
| Thrombosis-CLANC | 0 (0) | 0 (0) | 0 (0) | 1 (2.0) |
| Culture negative sepsis | 2 (1.5) | 4 (1.1) | 1 (1.9) | 0 (0) |
| No harm or still in use | 79 (59) | 319 (88) | 42 (81) | 43 (84) |
| Statistics presented as n (column %). CLABSI (Central Line Associated Blood Stream Infection), CLANC (Central Line Associated Non-CLABSI Complication) | | | | |
|  | | | | |
