# Supplementary material for: Central-line–associated bloodstream infections and central-line–associated non-CLABSI complications among pediatric oncology patients
Source: Infect Control Hosp Epidemiol. 2022 Apr 27;44(3):377–83. doi: 10.1017/ice.2022.91 (PMC10015264; doi:10.1017/ice.2022.91)
Supplement: Supplementary file 1 [file S0899823X22000915sup001.zip › S0899823X22000915supp002.docx]

| Supplemental Table 1. Central Line-level Patient Characteristics, Catheter Features, Placement Duration, and Complications for Central Lines (n=650) Inserted in Pediatric and Young Adult Oncology Patients | |
| --- | --- |
| Factor |  |
| Age at line placement (years), median [Q1, Q3] | 7 [2, 14] |
| Age at line placement, n (%) |  |
| < 1 year | 48 (7.4) |
| 1 – 3 years | 120 (18) |
| 3 – 12 years | 235 (36) |
| 12 years and up | 247 (38) |
| CVC type, n (%) |  |
| Tunneled | 168 (26) |
| Mediport | 375 (58) |
| Apheresis | 54 (8.3) |
| Non-tunneled | 53 (8.2) |
| Tunneled Status, n (%) |  |
| Not tunneled | 91 (14) |
| Tunneled | 559 (86) |
| Line Days, median [Q1, Q3] | 152 [45, 362] |
| Inpatient Line Days, median [Q1, Q3] | 17 [6, 38] |
| Outpatient Line Days, median [Q1, Q3] | 122 [16, 326] |
| Number of lumens, n (%) |  |
| 1 | 409 (63) |
| 2 | 210 (32) |
| 3 | 31 (4.8) |
| Lumen Days, median [Q1, Q3] | 198 [67, 454] |
| Inpatient Lumen Days, median [Q1, Q3] | 21 [6, 56] |
| Outpatient Lumen Days, median [Q1, Q3] | 160 [26, 402] |
| Total CLABSIs, n (%) |  |
| 0 | 551 (85) |
| 1 | 90 (14) |
| 2 | 7 (1.1) |
| 3 | 2 (0.31) |
| Number of Inpatient CLABSIs, n (%) |  |
| 0 | 612 (94) |
| 1 | 34 (5.2) |
| 2 | 4 (0.62) |
| Number of Outpatient CLABSIs, n (%) |  |
| 0 | 587 (90) |
| 1 | 58 (8.9) |
| 2 | 5 (0.77) |
| Total CLANCs, n (%) |  |
| 0 | 568 (87) |
| 1 | 82 (13) |
| Number of Inpatient CLANCs, n (%) |  |
| 0 | 611 (94) |
| 1 | 39 (6.0) |
| Number of Outpatient CLANCs, n (%) |  |
| 0 | 607 (93) |
| 1 | 43 (6.6) |
| Bone Marrow Transplant, n (%) |  |
| No | 608 (94) |
| Yes | 42 (6.5) |
| Statistics presented as Median [P25, P75], N (column %). CLABSI (Central Line Associated Blood Stream Infection), CLANC (Central Line Associated Non-CLABSI Complication), CVC (Central Venous Catheter), Lumen Days (Line Days x Number of Lumens) | |
