# Supplementary material for: Central-line–associated bloodstream infections and central-line–associated non-CLABSI complications among pediatric oncology patients
Source: Infect Control Hosp Epidemiol. 2022 Apr 27;44(3):377–83. doi: 10.1017/ice.2022.91 (PMC10015264; doi:10.1017/ice.2022.91)
Supplement: Supplementary file 1 [file S0899823X22000915sup001.zip › S0899823X22000915supp003.docx]

| Supplemental Table 6: Comparison of Patient-Level Incidence Rates and Risk Factors for MBI-CLABSIs in Pediatric and Young Adult Oncology Patients (n=366) | | | |
| --- | --- | --- | --- |
| Risk Factor | Comparison | Incidence risk ratio (95% CI) | P-value |
| Age at diagnosis | < 1 year vs older | 8.7 (2.6, 28.6) | <0.001 |
|  | Per 1 year older | 0.97 (0.90, 1.0) | 0.44 |
| Diagnosis | AML vs all others | 25.9 (9.5, 70.6) | <0.001 |
|  | AML vs Non-AML Leukemia/Lymphoma | 35.9 (11.0, 116.4) | <0.001 |
|  | AML vs brain tumors | 66.2 (12.3, 356.3) | <0.001 |
|  | All others vs brain tumors | 5.4 (0.63, 47.4) | 0.12 |
| Gender | Female vs male | 2.7 (1.1, 7.0) | 0.037 |
| Number of central lines | Per 1 additional line | 1.9 (1.4, 2.8) | <0.001 |
| AML (Acute Myeloid Leukemia), CLABSI (Central Line Associated Blood Stream Infection), CLANC (Central Line Associated Non-CLABSI Complication) | | | |
