# Supplementary material for: Central-line–associated bloodstream infections and central-line–associated non-CLABSI complications among pediatric oncology patients
Source: Infect Control Hosp Epidemiol. 2022 Apr 27;44(3):377–83. doi: 10.1017/ice.2022.91 (PMC10015264; doi:10.1017/ice.2022.91)
Supplement: Supplementary file 1 [file S0899823X22000915sup001.zip › S0899823X22000915supp004.docx]

| Supplemental Table 5. Comparison of First Line Incidence Rate Ratios and Risk Factors for All Events, CLABSIs and CLANCs in Pediatric and Young Adult Oncology Patients (n=366) | | | | | | | |
| --- | --- | --- | --- | --- | --- | --- | --- |
| Risk factor | Comparison | CLABSI Incidence rate ratio (95% CI) | P-value | CLANC Incidence rate ratio (95% CI) | P-value | All Events Incidence rate ratio (95% CI) | P-value |
| Age at diagnosis | Per 1 year older | 0.96 (0.90, 1.03) | 0.31 | 0.87 (0.77, 0.97) | 0.015 | 0.92 (0.87, 0.99) | 0.017 |
|  | Age < 1 year vs older | 6.3 (1.6, 24.9) | 0.008 | 49.3 (6.8, 355.0) | <0.001 | 18.0 (5.0, 64.6) | <0.001 |
| Age at placement | Per 1 year older | 0.98 (0.91, 1.05) | 0.54 | 0.86 (0.77, 0.97) | 0.016 | 0.94 (0.88, 1.00) | 0.053 |
|  | Age < 1 year vs older | 7.0 (1.7, 28.5) | 0.006 | 46.0 (5.5, 386.9) | <0.001 | 18.3 (4.7, 70.7) | <0.001 |
| Gender | Female vs male | 1.2 (0.47, 2.9) | 0.74 | 3.4 (0.74, 15.4) | 0.11 | 1.7 (0.72, 3.8) | 0.24 |
| CVC type | > 1 lumen vs 1 lumen | 5.4 (2.4, 12.5) | <0.001 | 2.9 (0.58, 14.8) | 0.19 | 4.7 (2.1, 10.6) | <0.001 |
|  | Not tunneled vs tunneled | 7.6 (1.7, 33.2) | 0.007 | 7.5 (0.99, 56.5) | 0.051 | 9.1 (2.6, 32.1) | <0.001 |
|  | Non-mediport vs mediport | 8.7 (4.3, 17.7) | <0.001 | 24.3 (8.0, 74.3) | <0.001 | 12.4 (6.5, 23.4) | <0.001 |
|  | Non-tunneled vs mediport | 7.7 (1.4, 41.9) | 0.018 | 44.7 (7.7, 260.0) | <0.001 | 21.4 (6.2, 73.4) | <0.001 |
|  | Tunneled vs mediport | 7.6 (3.6, 16.2) | <0.001 | 22.1 (6.4, 76.5) | <0.001 | 10.6 (5.2, 21.4) | <0.001 |
|  | Apheresis catheter vs mediport | 24.0 (4.9, 117.2) | <0.001 | 5.4 (0.22, 132.7) | 0.3 | 14.5 (3.0, 70.9) | <0.001 |
| Diagnosis | AML vs all others | 5.7 (1.3, 25.4) | 0.023 | 0.96 (0.06, 16.8) | 0.98 | 2.8 (0.62, 12.5) | 0.18 |
|  | AML vs Non-AML Leukemia/Lymphoma | 5.4 (1.03, 28.2) | 0.046 | 0.85 (0.05, 15.1) | 0.91 | 2.1 (0.43, 10.6) | 0.36 |
|  | AML vs brain tumors | 8.0 (1.4, 45.3) | 0.018 | 2.0 (0.09, 48.2) | 0.66 | 4.1 (0.71, 24.1) | 0.11 |
|  | All others vs brain tumors | 0.64 (0.04, 10.3) | 0.75 | 14.7 (0.54, 401.1) | 0.11 | 5.6 (0.63, 49.6) | 0.12 |
| Incidence Rates are per 1000 line Days. AML (Acute Myeloid Leukemia), CLABSI (Central Line Associated Blood Stream Infection), CLANC (Central Line Associated Non-CLABSI Complication), CVC (Central Venous Catheter) | | | | | | | |
