# Supplementary material for: Central-line–associated bloodstream infections and central-line–associated non-CLABSI complications among pediatric oncology patients
Source: Infect Control Hosp Epidemiol. 2022 Apr 27;44(3):377–83. doi: 10.1017/ice.2022.91 (PMC10015264; doi:10.1017/ice.2022.91)
Supplement: Supplementary file 1 [file S0899823X22000915sup001.zip › S0899823X22000915supp005.docx]

| Supplemental Table 2. Patient-Level risk factors and Incidence Rate Ratios for CLABSIs, CLANCs, and All Events in Pediatric and Young Adult Oncology Patients (n=366) | | | | | | | |
| --- | --- | --- | --- | --- | --- | --- | --- |
| Risk factor | Comparison | CLABSI Incidence rate ratio (95% CI) | P-value | CLANC Incidence rate ratio (95% CI) | P-value | All Events Incidence rate ratio (95% CI) | P-value |
| Age at diagnosis | < 1 year vs older | 4.7 (2.0, 10.9) | <0.001 | 13.8 (7.3, 25.9) | <0.001 | 7.6 (3.8, 15.2) | <0.001 |
|  | Per 1 year older | 0.97 (0.92, 1.02) | 0.18 | 0.89 (0.84, 0.94) | <0.001 | 0.94 (0.90, 0.97) | 0.001 |
| Diagnosis | AML vs all others | 6.8 (2.6, 17.8) | <0.001 | 4.7 (1.5, 14.7) | 0.008 | 5.9 (2.4, 14.1) | <0.001 |
|  | AML vs Non-AML Leukemia/Lymphoma | 8.3 (3.1, 22.3) | <0.001 | 5.5 (1.8, 17.1) | 0.003 | 7.2 (2.9, 17.6) | <0.001 |
|  | AML vs brain tumors | 11.4 (3.7, 34.7) | <0.001 | 8.5 (2.3, 31.7) | 0.001 | 9.9 (3.6, 27.0) | <0.001 |
|  | All others vs brain tumors | 1.9 (0.43, 8.6) | 0.39 | 12.2 (3.0, 48.6) | <0.001 | 5.9 (1.9, 18.6) | 0.003 |
| Gender | Female vs male | 1.5 (0.85, 2.7) | 0.16 | 1.7 (0.87, 3.4) | 0.12 | 1.7 (1.00, 2.8) | 0.05 |
| Number of central lines | Per 1 additional central line | 1.8 (1.5, 2.1) | <0.001 | 1.8 (1.5, 2.2) | <0.001 | 1.9 (1.6, 2.2) | <0.001 |

CLABSI (Central line associated blood stream infection), CLANC (Central line associated non-CLABSI) complication), AML (Acute Myeloid Leukemia)
