# Supplementary material for: Central-line–associated bloodstream infections and central-line–associated non-CLABSI complications among pediatric oncology patients
Source: Infect Control Hosp Epidemiol. 2022 Apr 27;44(3):377–83. doi: 10.1017/ice.2022.91 (PMC10015264; doi:10.1017/ice.2022.91)
Supplement: Supplementary file 1 [file S0899823X22000915sup001.zip › S0899823X22000915supp006.docx]

| Supplemental Table 7. Comparison of Incidence Rate Ratios and Risk Factors for MBI-CLABSIs for All Central Lines Treated Independently (N=650) | | | |
| --- | --- | --- | --- |
| Risk factor | Comparison | Incidence risk ratio (95% CI) | P-value |
| Age at diagnosis | Per 1 year older | 0.96 (0.89, 1.0) | 0.36 |
|  | < 1 year vs older | 7.3 (1.8, 29.2) | 0.005 |
| Age at placement | Per 1 year older | 0.96 (0.88, 1.0) | 0.32 |
|  | Age < 1 year vs older | 5.0 (0.82, 30.1) | 0.081 |
| Diagnosis | AML vs other diagnoses | 22.0 (6.6, 73.0) | <0.001 |
|  | AML vs Non-AML Leukemia/Lymphoma | 30.5 (7.9, 118.4) | <0.001 |
|  | AML vs brain tumors | 72.6 (11.9, 443.1) | <0.001 |
|  | All others vs brain tumors | 6.7 (0.62, 71.7) | 0.12 |
| Gender | Female vs male | 2.4 (0.77, 7.5) | 0.13 |
| Central line insertion rank order | Per 1 additional line | 1.2 (0.84, 1.6) | 0.35 |
| CVC type | > 1 lumen vs 1 lumen | 18.9 (7.9, 45.4) | <0.001 |
|  | Not tunneled vs tunneled | 0.35 (0.024, 5.1) | 0.44 |
|  | Non-mediport vs mediport | 24.9 (10.4, 60.0) | <0.001 |
|  | Non-tunneled^*^ vs mediport | 0 | -- |
|  | Tunneled vs mediport | 30.3 (12.4, 74.2) | <0.001 |
|  | Apheresis catheter vs mediport | 13.8 (2.3, 83.4) | 0.004 |
| *There were no MBI-CLABSIs in non-tunneled lines. AML (Acute Myeloid Leukemia), CLABSI (Central Line Associated Blood Stream Infection), CVC (Central Venous Catheter), MBI (Mucosal Barrier Injury-Associated) | | | |
