# Supplementary material for: Central-line–associated bloodstream infections and central-line–associated non-CLABSI complications among pediatric oncology patients
Source: Infect Control Hosp Epidemiol. 2022 Apr 27;44(3):377–83. doi: 10.1017/ice.2022.91 (PMC10015264; doi:10.1017/ice.2022.91)
Supplement: Supplementary file 1 [file S0899823X22000915sup001.zip › S0899823X22000915supp007.docx]

|  | | | | |
| --- | --- | --- | --- | --- |
| Supplemental Table 3. Indication for Central Line Removal for Pediatric Oncology Patients < 1 year at Age of Placement Stratified by Line Type | | | | |
| Factor | Tunneled (n=33) | Mediport (n=11) | Apheresis (n=2) | Non-tunneled (n=2) |
| Reason for line removal |  |  |  |  |
| CLABSI | 3 (9.1) | 1 (9.1) | 0 (0) | 0 (0) |
| Breakage-CLANC | 2 (6.1) | 0 (0) | 1 (50) | 0 (0) |
| Contamination-CLANC | 1 (3.0) | 0 (0) | 0 (0) | 0 (0) |
| Dislodgement-CLANC | 6 (18) | 0 (0) | 0 (0) | 0 (0) |
| Exit Site Problem-CLANC | 0 (0) | 1 (9.1) | 0 (0) | 0 (0) |
| Malfunction-CLANC | 1 (3.0) | 2 (18) | 1 (50) | 0 (0) |
| Malposition-CLANC | 5 (15) | 1 (9.1) | 0 (0) | 1 (50) |
| Relocation-CLANC | 1 (3.0) | 0 (0) | 0 (0) | 0 (0) |
| No harm or still in use | 14 (42) | 6 (55) | 0 (0) | 1 (50) |
| Statistics presented as n (column %). CLABSI (Central Line Associated Blood Stream Infection), CLANC (Central Line Associated Non-CLABSI Complication) | | | | |
